# Platelet-Rich Fibrin Promotes Osteoblast Recruitment-Associated Periodontal Regeneration via Macrophage Polarization

**DOI:** 10.1155/sci/9912405

**Published:** 2025-08-15

**Authors:** Hudi Xu, Huan Jing, Richard J. Miron, Yulan Wang, Dagang Xu, Xiaoxin Zhang

**Affiliations:** ^1^Department of Oral Implantology, Stomatology Center, Peking University Shenzhen Hospital, Shenzhen, Guangdong Province, China; ^2^Department of Endodontics, Stomatology Center, Peking University Shenzhen Hospital, Shenzhen, Guangdong Province, China; ^3^Department of Periodontology, University of Bern, Bern, Switzerland; ^4^The State Key Laboratory Breeding Base of Basic Science of Stomatology (Hubei-MOST) and Key Laboratory of Oral Biomedicine Ministry of Education, School and Hospital of Stomatology, Wuhan University, Wuhan, Hubei, China; ^5^School of Stomatology, Hubei University of Science and Technology, Xianning, Hubei, China

**Keywords:** macrophage, osteoblast, periodontal regeneration, platelet-rich fibrin

## Abstract

**Aims:** Inflammation is a key process involved in the early stages of periodontal regeneration, where immune cells are responsible for the recruitment of osteoblast to facilitate periodontal regeneration. The aim of the present study was to explore the effect of platelet-rich fibrin (PRF) on macrophage polarization, and thereafter to investigate its effect on osteoblast recruitment to enhance early-stage periodontal regeneration.

**Materials and Methods:** The extracted liquids of PRF, produced using fixed-angled and horizontal centrifugation protocols, were utilized to stimulate Thp1 to study macrophage proliferation and polarization. Thereafter, the supernatants of Thp1 were collected and utilized to stimulate the migration of human bone marrow osteoblasts, to investigate the recruitment of osteoblast via macrophage polarization.

**Results:** PRF stimulated the proliferation and recruitment of macrophages, with horizontal centrifugation protocols demonstrating significantly greater potential when compared to fixed-angled. Furthermore, PRF was able to enhance the recruitment of osteoblast via macrophage polarization, with horizontal platelet-rich fibrin (H-PRF) demonstrating the most significant increase.

**Conclusion:** The present study explored a promising mechanism of the periodontal regeneration function of PRF, by inducing macrophage polarization, thereby enhancing osteoblast recruitment, with horizontal centrifugation significantly improving these findings.

**Summary**

• Scientific rationale for the study: Platelet-Rich fibrin (PRF) prepared by centrifugation is widely used in various fields of medicine for improved periodontal regeneration benefitted by its ability to enhance angiogenesis and wound healing. Therefore, the mechanism of the effect of PRF on immune cells and the outcome of horizontal centrifuge utilization remains unclear.
• Principal findings: A promising mechanism of the periodontal regeneration function of PRF by inducing macrophage polarization, thereby enhancing osteoblasts recruitment has been explored. It also revealed that horizontal centrifugation significantly improved these findings.
• Practical implications: The utilization of horizontal centrifuge on PRF production could be a promising protocol to present better periodontal regeneration.

## 1. Introduction

Periodontal regeneration and wound healing processes require interaction between different periodontals, including paracrine and autocrine interactions [1]. Proper differentiation of various osteoblast populations is critical for the smooth progression of periodontal regeneration [2]. Osteoblasts, derived from mesenchymal stem cells (MSCs), play a key role in bone regeneration [3]. During early healing, MSCs differentiate into chondrocytes that form a cartilage matrix, creating a soft callus later mineralized by osteoblasts through the secretion of type I collagen and bone matrix proteins [4, 5]. In the subperiosteal and surrounding soft tissue areas, recruited MSCs and preosteoblasts differentiate into osteoblasts that directly form hard callus by producing extracellular matrix and bone mineral crystals [6]. Osteoblasts also promote cell adhesion, proliferation, and differentiation. Their development is regulated by BMP signaling through Smad1/5/8 and RUNX2 pathways, and some mature into osteocytes once embedded in the bone matrix [7]. Proper osteoblast function, including apoptosis, migration, and differentiation, is essential for bone homeostasis, and its disruption is linked to diseases, such as osteoporosis, osteopetrosis, and Paget's disease [8].

Inflammation is naturally a key process involved in the early stages of soft periodontal and bone healing [9], where immune cells recruit osteoblasts that participate in their regulation during bone regeneration [10]. During inflammation, cytokines secreted by macrophages—such as tumor necrosis factor-α (TNF-α) and interferon gamma (IFN-γ)—are responsible for the early inflammatory response capable of recruiting further cells. These macrophages are typically classified, as type I macrophages (M1s). Considered an important immune effector cell of the innate immune system, macrophages not only play important roles as a first line of microbe defense but also play a decisive role in periodontal repair and regeneration [11–13]. They secrete various chemokines (such as MCP-1 and MIP-1α) that regulate osteoblast recruitment during the initial inflammatory phase, which then participate in osteogenesis and bone matrix formation [12, 14]. In order to enhance the recruitment of osteoblasts, the early polarization of macrophages towards their M1 phenotype to create a suitable immune environment for early-stage periodontal repair is critical.

Platelet-rich fibrin (PRF), a second-generation platelet concentrate prepared by centrifugation without anticoagulation, is widely used in various fields of medicine for improved periodontal regeneration, benefitted by its ability to enhance angiogenesis and wound healing [15–17]. PRF differs from its precursor, platelet-rich plasma (PRP), in that it contains a higher concentration of leukocytes embedded within its fibrin network, with multiple cytokines secreted over a 10–14-day period [18]. These white blood cells, along with their release of growth factors, are major players in the adaptation of the immune environment [19, 20].

Several protocols have been proposed for the production of PRF. Traditional PRF, mainly referred to as leukocyte-platelet-rich fibrin (L-PRF), most commonly utilizes a centrifugation protocol of 700 g for 12 min [21]. More recently, advanced platelet-rich fibrin (A-PRF) was developed using lower centrifugation force and time, aiming to further concentrate cells in the upper plasma-rich layer by utilizing a protocol of 200 g for 8 min [20, 22]. Today, the majority of devices used to produce PRF rely on fixed-angled centrifugation. Recently, our group has shown that horizontal centrifugation is better able to separate blood cell layers and accumulate platelets and leukocytes more efficiently [23, 24].

Chronic inflammation is a pathological condition in which mesenchymal lineage cells become a significant source of inflammatory mediators. PRF has demonstrated notable anti-inflammatory effects, particularly in macrophages, where it promotes polarization from a pro-inflammatory M1 to an anti-inflammatory M2 phenotype. The present study shows that both liquid and solid PRF exert anti-inflammatory activity in murine mesenchymal cells [25]. However, in human cells, the response appears more complex. For example, in gingival fibroblasts, PRF membrane lysates and PRF serum both increase chemokine expression, though membrane lysates induce a broader and more complex inflammatory profile. These findings suggest that, while PRF has anti-inflammatory properties in certain contexts, it may also provoke an inflammatory response depending on cell type and preparation [26, 27].

Research on the effect of PRF on macrophage polarization is very limited. One study indicated that PRF can induce macrophage polarization towards the M2 phenotype, but this research was based on Wistar rats and RAW cells [28]. The aim of the present study was to explore whether PRF could induce osteoblast recruitment through macrophage polarization. Three different centrifugation methods were utilized to investigate whether PRF produced via various centrifugation devices could impact macrophage polarization and osteoblast recruitment.

## 2. Materials and Methods

### 2.1. Preparation of L- PRF, A-PRF, and H-PRF

The collection of whole blood sample was performed with the informed and written consent of the subjects. Blood samples were collected with the informed consent from six authors. The experiments were undertaken with the understanding and written consent of each subject and according to the ethical standards of the World Medical Association Declaration of Helsinki and the additional requirements. No ethical approval was considered required for this study since all blood was collected from authors included in this study, and human samples were not identified, as previously described [29]. Authors that denoted blood were healthy, nonsmoking, and not taking any medications volunteers between the ages of 20 and 40 years old without any systematic diseases that may affect fibrin clot formation and structure. According to previous research that following diseases had been excluded: abnormal concentration of factor XIII and thrombin in plasma, abnormal in platelet activation, hyperglycemia, hyper-homocysteinemia, oxidative stress, and cigaret smoking [30]. L-PRF was prepared with an angled centrifuge (IntraSpin centrifuge, IntraLock, Boca Raton, Florida) using 9 mL plastic silica-coated tubes drawn from volunteers without anticoagulant at 700 g (RCF-max) for 12 min at room temperature, immediately. A-PRF was produced using a fixed-angle centrifuge (process for PRF, Nice, France) at 200 g for 8 min using 10 mL silicone-coated glass tubes (process for PRF). For horizontal platelet-rich fibrin (H-PRF), the blood was centrifuged in a horizontal centrifuge (Cence 550I centrifuge, China) at 700 g for 8 min. The collected whole blood and PRF were then transferred to a 6-well plate and incubated with 5 mL of cell culture medium (RPMI; HyClone, Thermo) in a 37°C incubator with 5% CO_2_. After 1 day, the medium was collected as conditioned media (CM) and normalized by their weight then utilized in future experiments using 20% CM as previously described [1].

### 2.2. Scanning Electron Microscopy (SEM)

Obtained L-PRF, A-PRF, and H-PRF were immediately fixed in 2.5% glutaraldehyde for 24 h at 4°C. Then L-PRF, A-PRF, and H-PRF were dehydrated in an order of 25%, 50%, 75%, 90%, and 100% ethanol solutions each for 5 min. After dehydrate using graded ethanol, PRF was processed by critical point drying then coated with 20 nm gold for surface roughing treatments. Finally, L-PRF, A-PRF, and H-PRF were examined using field emission scanning electron microscope (NOVA NANOSEM430, FEI, Netherlands). Pictures were taken using 5k–10k× magnifications, 15–20 kV. Each experiment was performed three times independently.

### 2.3. Cell Culture of Thp1 and hFOB

Thp1, a widely used human leukemia monocytic cell line, was cultured using RPMI cell culture media (HyClone, Thermo) with 10% fetal bovine serum (FBS; Gibco, Life Technologies Corporation). A density of 1 × 10^6^ cells/ml of Thp1 was first induced to macrophages by stimulation using 100 ng/mL phorbyl myristate acetate (PMA, Sigma) for 48 h. Then the culture medium was changed to whole blood, L-PRF, A-PRF, or H-PRF CM for 24 h.

hFOB 1.19 were utilized in this study (Human Fetal Osteoblasts), and the cells were cultured in DMEM medium with 10% fetal bovine serum (FBS; Gibco, Life Technologies Corporation). During CM experiments, hFOB 1.19 cells were additionally cultured with the supernatants of Thp1 cells. The control group was cultured using only DMEM medium, and the other groups were cultured with half the supernatants of Thp1 treated with either whole blood or H-PRF CM.

### 2.4. Transwell Migration Assay

The migration of Thp1 was performed using the transwell chamber with a pore size of 3 μm (Corning coster, Corning). The extracted liquids of whole blood, A-PRF, L-PRF, or H-PRF with 10% FBS (500 uL/per well) was filled into the lower wells. After starving the cells for 24 h in RPMI containing 0.5% FBS, Thp1 cells were seeded in the upper transwell chamber (10^4^ cells/100 uL/per well) and cultured in a 37° C incubator with 5% CO_2_ for 24 h. Thp1 cells that migrated to the lower layer were counted using a cell counter (Vi-CELL XR, Beckman, USA).

The migration of hFOB was performed using a transwell chamber with a pore size of 8 μm (Corning coster, Corning). After 24 h incubation, hFOB were fixed by 4% formaldehyde (FA) for 5 min. Then hFOB that had migrated through the transwell were stained by 0.5% crystal violet (Amresco, Solon) for 15 min. After staining, the chambers were washed three times using phosphate-buffered saline (PBS). The images of all stained migrated cells were then captured and counted under a microscope (Olympus, Japan. 5 fields/well, × 200 magnification). Each experiment was performed three times independently.

### 2.5. Scratch Wound Healing Assay

hFOBs (5× 10^5^ cells per well) were seeded into 6-well plates. After 8 h of incubation, a 10 μL pipette tip was used to scratch the cell layer. And the culture media was changed to whole blood, H-PRF and the supernatants of Thp1 treated with whole blood and H-PRF extracted liquids. Images of the initial state of hFOBs were immediately captured using a light microscope (Olympus, Japan) and captured again after 6, 12, and 24 h [31, 32].

Reference points were drawn in order to find the location of the scratch and obtain the same field of view. Each experiment was performed three times independently.

### 2.6. Proliferation Assay

With the stimulation of culture medium and the extracted liquids of whole blood, A-PRF, L-PRF, or H-PRF in RPMI with 10% FBS (500 μL/per well), 10^3^ cells/well Thp1 were first seeded in 96-well plates. On days 1, 3, and 5, medium was removed, replaced with Cell Counting Kit-8 (Dojindo, Japan) and incubated for 1.5 h. Then the O.D. absorbance was detected at 450 nm using a microplate reader (PowerWave XS2, BioTek, Winooski, VT, USA). Each experiment was performed three times independently.

### 2.7. Real-Time PCR for Differentiation Markers

Thp1 was first differentiated towards the macrophage lineage using 100 ng/mL phorbyl myristate acetate (PMA, Sigma) at a density of 1 × 10^6^ cells/ml for 48 h. Then the culture medium was changed to whole blood, L-PRF, A-PRF, and H-PRF extracted liquids for 24 h. After 24 h stimulation, total mRNA from cells was isolated using AxyPrepTM Multisource Total RNA Miniprep Kit (AXYGEN, Union City, California, USA). Then, the concentration of RNA was determined by a NanoDrop 2000 UV–Vis Spectrophotometer and 1 μg RNA were reverse transcribed to cDNA using a cDNA Synthesis Kit (GeneCopoeia, Maryland, USA). 10 μl final reaction volume was performed using All-in-One TM qPCR Mix Kit (GeneCopoeia, Maryland, USA), then detected on a Real-Time PCR Detection System (Biorad). GAPDH was used to normalize the amount of mRNA of interest. Sequences of primers utilized are listed in Table 1. Each experiments was performed three times independently.

### 2.8. Statistical Analysis

All data analysis was quantified using SPSS software (IBM, USA). The significance was reported at *p* < 0.05. The data was calculated using the ^*ΔΔ*^Ct method and the statistical significance of differences between different groups were examined by one-way ANOVA and *t*-test. (*⁣*^*∗*^*p* < 0.05, *⁣*^*∗∗*^*p* < 0.01, *⁣*^*∗∗∗*^*p* < 0.001)

## 3. Results

### 3.1. Morphological Appearances of A-PRF, L-PRF, and H-PRF

**Figure 1:**
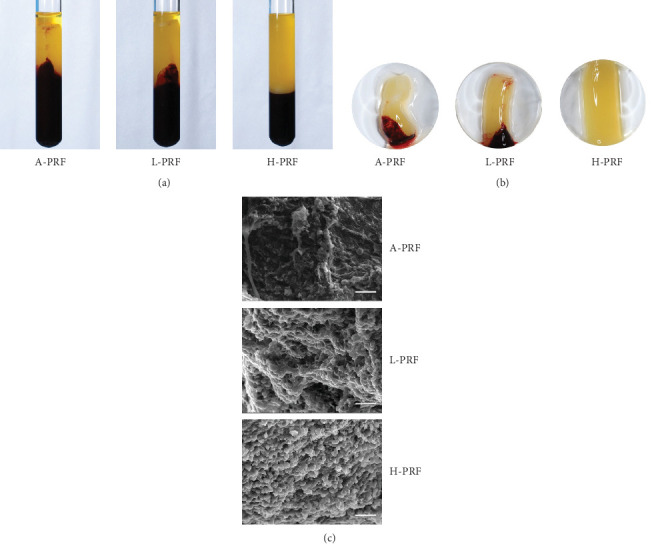
Characterization of A-PRF, L-PRF, and H-PRF. (a, b) The morphology and size of A-PRF, L-PRF, and H-PRF after centrifugation using the manufacturers recommended centrifugation protocols and tubes. (c) Scanning electron microscopy (SEM) of A-PRF, L-PRF, and H-PRF clots. Scale bar = 20 μm.

Recent studies by our group demonstrated that horizontal centrifugation significantly increased both the number and concentration of platelets and leukocytes in PRF when compared to fixed-angle centrifuge (InstraSpin, Process for PRF) [24]. When centrifugation was carried out, the PRF clots produced via horizontal centrifugation were larger and more noticeably, the separation between the red blood cell layer was much more distinct in H-PRF when compared to the other two fixed-angled groups (Figure 1a,b). When the morphological features of A-PRF, L-PRF, and H-PRF clots were investigated by SEM, it was observed that more visible cells were trapped in the fibrin mesh in the H-PRF group compared to either the fixed-angle centrifugation utilized to produce A-PRF and L-PRF.

### 3.2. The Effect of L-PRF, A-PRF, and H-PRF on Macrophages Migration and Proliferation

**Figure 2:**
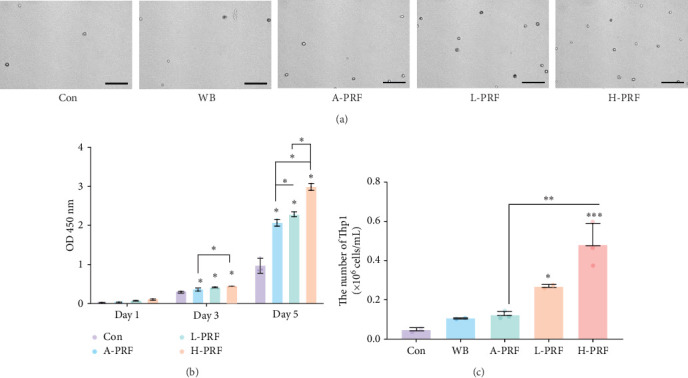
Migration and proliferation assays of Thp1 cells in response to PRF. (a, c)The number of migrated cells (10^4^ per well) following use of a transwell migration assay analyzed after culture for 12 h. (b) CCK-8 assay analyzed the proliferation ratio of the various PRF groups up to 5 days. *⁣*^*∗*^*p* < 0.05; *⁣*^*∗∗*^*p* < 0.01; *⁣*^*∗∗∗*^*p* < 0.001; NS: not statistically significant vs. control group. CCK-8, cell counting kit-8. Scale bar = 100 μm.

In order to focus on the cytokines that were produced by leukocytes and platelets from PRF, the culture medium was collected from each of the L-PRF, A-PRF, and H-PRF groups and compared to whole blood. A CCK-8 assay was performed to test cell proliferation rates (Figure 2b). It was found that cell numbers were significantly higher in three PRF treated groups when compared to whole blood on days 3 and 5 (*p* < 0.05). No significant differences were reported between the L-PRF, A-PRF, and H-PRF groups. To examine the effects of L-PRF, A-PRF, and H-PRF on the migration of Thp1 cells, a transwell migration assay was performed (Figure 2a,c). It was observed that a significantly elevated number of Thp1 cells was found in the H-PRF group when compared to either the L-PRF and A-PRF groups.

### 3.3. Effect of L-PRF, A-PRF, and H-PRF on Macrophage Polarization

**Figure 3:**
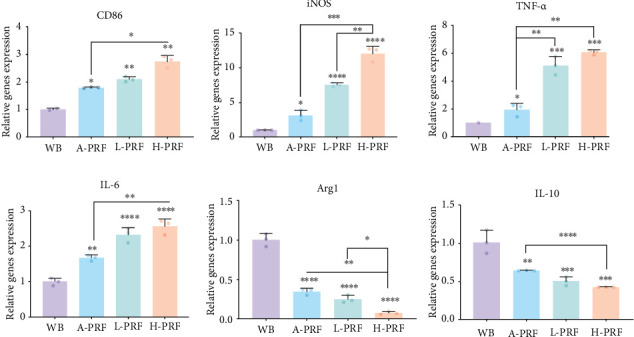
A-PRF, L-PRF, and H-PRF facilitates M1 macrophage polarization. Relative mRNA expression of CD86, iNOS, TNF- α, IL-6, ARG1, and IL-10, of Thp1 cells after stimulating by whole blood (WB), A-PRF, L-PRF, and H-PRF extract. *⁣*^*∗*^*p* < 0.05, *⁣*^*∗∗*^*p* < 0.01, *⁣*^*∗∗∗*^*p* < 0.001, *⁣*^*∗∗∗∗*^*p* < 0.0001.

The early pro-inflammatory ability of PRF was investigated on initial M1 (TNF-a, CD86, IL-6, and iNOS) and M2 (IL-10 and ARG1) associated markers and cytokines. Stimulation of Thp1 cells with PRF extracted liquids resulted in a shift toward the M1 phenotype with a higher expression of M1 markers (Figure 3). The H-PRF treated group demonstrated the greatest shift in macrophage polarization.

### 3.4. L- PRF, A-PRF, and H-PRF Induced the Recruitment of Bone Marrow Osteoblaststhrough Macrophage Polarization

**Figure 4:**
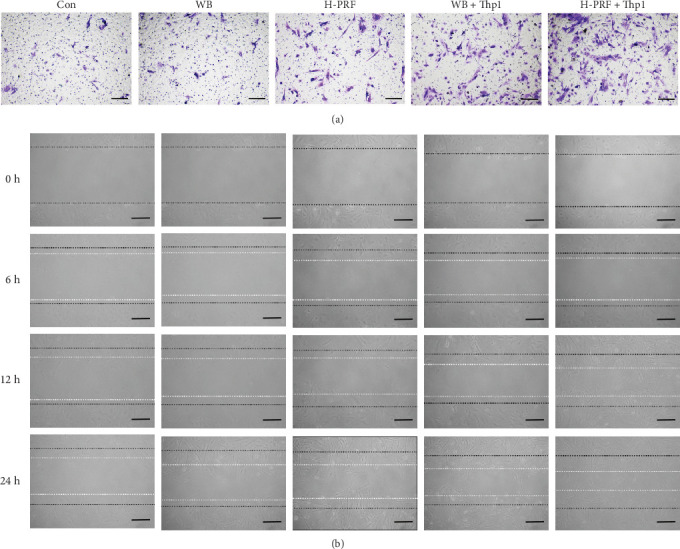
Migration assay and scratch assay of hFOB in response to stimulation of Thp1 supernatant. (a) Images of the transwell migration assay. Note that more cells found in the H-PRF group. (b) Images from the scratch wound-healing assay. The black line depicts the scratches made at time point zero, whereas the white line represents the line at depicted time point. Scale bar = 100 μm.

**Figure 5:**
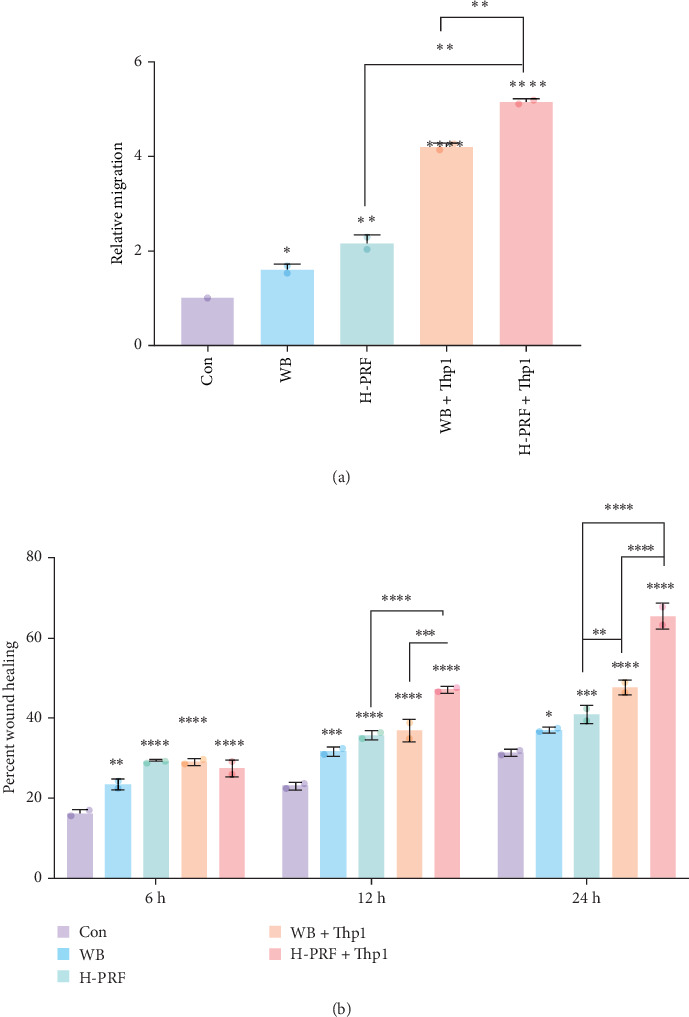
Quantitative data from the migration assay of hFOB in response to stimulation of Thp1 supernatant. (a) Quantitative data from the transwell migration assay. The relative migration was measured using imageJ software. (b) Quantitative data from the scratch wound healing assay depicting the percentage of wound healing measured using Photoshop software. Percent wound healing = (D0h − D24h)/D0h x 100, D0h is the area at the start of the scratch and D24h is the areas after 6, 12, and 24 h. *⁣*^*∗*^*p* < 0.05, *⁣*^*∗∗*^*p* < 0.01, *⁣*^*∗∗∗*^*p* < 0.001, *⁣*^*∗∗∗∗*^*p* < 0.0001.

As the previous results showed, the macrophage inducing ablity of H-PRF was significant higher than A-PRF and L-PRF. H-PRF was chosen to investigate further the effect of PRF on osteoblastsmigration through macrophages polarization. Compared to the control group and the CM from Thp1 cells cultured with whole blood (WB + Thp1 group), H-PRF (H-PRF + Thp1 group), the supernatants of Thp1 treated with whole blood and H-PRF CM significantly upregulated bone marrow osteoblastsmigration (Figures 4 and 5). The results indicate that the effect of PRF on osteoblastsmigration is partly regulated through macrophages polarization. Furthermore, the number of migrated bone marrow osteoblastsand the migrated ability was significant higher in the H-PRF + Thp1 group comparing to WB + Thp1 group, which confirm the periodontal regeneration ability of PRF.

## 4. Discussion

PRF consists of a fibrin matrix with entrapment of platelets, leukocytes, and cytokines. The fibrin mesh has previously been shown to favor the release of growth factors over a slower and extended period of time favoring periodontal regeneration of either hard or soft periodontals [33]. Previous research has shown that PRF favors primarily soft periodontal regeneration and also possesses some antibacterial function [34, 35]. As such, L-PRF was initially described as a first protocol utilized for the production of PRF utilizing a 700 g for 12 min centrifugation protocol, whereas more recent research has demonstrated that a slower spin cycle (A-PRF) was developed to favor a higher release of growth factors using a 200 g for 8 min protocol [20]. Previous studies have demonstrated that A-PRF have a more porous structure and greater interfacial space compared to L-PRF [24]. In this study, we also compared the effects of A-PRF and L-PRF on macrophage polarization, further verifying that A-PRF can release more and more sustained growth factors.

Both A-PRF and L-PRF use a fixed-angle centrifuge to perform the centrifugation process, more recently, our group demonstrated that horizontal centrifuges significantly increased both the number and concentration of platelets and leukocytes in PRF matrix when compared to angled centrifuges (InstraSpin, Process for PRF) [24]. First, with the tube completely horizontal produced by a swing out bucket, the greatest range between the minimum and maximum g-force range was observed. This larger g-force gradient between the RCF-min and RCF-max leads to improved cells separation. Second, fixed-angled centrifugation brings about more cell trauma since the centrifugal force is directed against the 30–45° tube wall whereas horizontal centrifugation does not have such interactions [24]. In the present study, it was reported that by utilizing horizontal centrifugation, the number of inflammatory cells and platelets was visibly increased which complements well with previous data [24]. Therefore, in this study, in order to further study the general applicability of PRF to macrophages, and to explore the biological effects of H-PRF on the number advantages of leukocytes, platelets, and growth factors, A-PRF, L-PRF, and H-PRF, all three PRF preparation protocols are used to stimulate macrophages. Macrophage polarization results of H-PRF also show more obvious biological effects. H-PRF can better promote macrophage appreciation, migration, and differentiation towards M1, which is conducive to inflammation and antibacterial in the early stage.

Considered an important immune effector cell of the innate immune system, macrophages not only play important roles as a first line of defense versus incoming microbes but also play a decisive role in periodontal repair and adaptive immune responses [36]. In addition to participating in the production of numerous growth factors, macrophages also play an important role in periodontal remodeling and metabolic function [37, 38]. In the present study, it was observed that macrophage proliferation and migration was highest in the H-PRF group, given the advantages of horizontal centrifugation in the number of leukocytes, platelets, and growth factors in previous studies. We speculate that several cytokines contained or released by PRF may be essential for the proliferation and migration of macrophages, such as macrophage colony-stimulating factor (M-CSF) and granulocyte-macrophage colony stimulating factor (GM-CSF). M-CSF and GM-CSF can be released by cells trapped in PRF, such as monocytes or neutrophils. M-CSF, which is also known as colony-stimulating factor 1 (CSF-1) is necessity for macrophage development, proliferation, morphology, and function [39, 40]. GM-CSF, which is also known as CSF-2, can promote the proliferation of peritoneal macrophages in vivo [41]. Furthermore, GM-CSF is essential for inducing g-collected monocytes which can contribute significantly to resident macrophage repertoires [42, 43].

Macrophages have been classified into two major phenotypes on the basis of multiple researchers, according to their type 1 helper T-cell (Th1)/type 2 helper T-cell (Th2) polarization [44–46]. The introduction of the Th1/Th2 paradigm and the significance of the inflammatory and cytotoxic nature of macrophages produced the concept that cytokines secreted by Th1 cells, such as IFN-γ and TNF-a, induced macrophage activation. These macrophages were then termed proinflammatory, or usually classified as type I macrophages (M1s) [47]. M1 macrophages possess potent microbicidal properties and support IL-12 mediated Th1 responses, which are essential for early periodontal regeneration [48]. It is suggested that with a greater number of growth factors released from more cells produced on a horizontal centrifuge, the induction and polarization of macrophages is also significantly induced. The results show that by promoting the value-added migration of macrophages and M1 polarization, the recruitment of osteoblasts has also been significantly promoted, providing new ideas for the mechanism of PRF to promote periodontal regeneration.

For recruitment of osteoblasts, macrophages gathered around PRF are demonstrated to produce multiple chemokines, for instance, chemoattractants exhibit selective effects on osteoblasts and MSCs. For instance, TGF-β1 promotes osteoblast migration in vitro but not that of MSCs, whereas BMP4 has the opposite effect [49]. However, in vivo studies suggest that chemotactic responses to multifunctional growth factors like TGF-β1 may be modulated by the local bone microenvironment [50]. Osteoblast migration is most commonly studied using cells cultured from bone in combination with assays, such as the Boyden chamber. To date, 27 osteoblasts chemoattractants have been identified, including CXCL16, IL-4, and IL-13 [51, 52]. Inflammatory cytokines, such as TNF-α, IFN-γ, TGF-β1, and IL-17 have also been shown to enhance BMP-2 secretion, further influencing osteoblast behavior. Additionally, osteoblast migration can be inhibited by chemorepellents, like leukemia inhibitory factor (LIF) and IL-1β, which block responses to specific chemoattractants without directly repelling the cells [53]. To date, three such chemorepellents have been identified.

For further research, the mechanism of chemokines produced by macrophages gathered around PRF inducing osteoblasts migration should be investigated.

## 5. Conclusions

The present study explored a promising mechanism of the bone regeneration function of PRF induced macrophage polarization and enhancement of osteoblasts recruitment. It also revealed that the production of PRF using horizontal centrifugation was significantly superior at promoting osteoblasts recruitment and in vitro periodontal regeneration assays.

## Figures and Tables

**Table 1:** The primer sequences used in RT-PCR.

Gene	Organism	Primer sequence
*IL-10*	*Homo sapiens*	Forward: 5′-GAGAAGCATGGCCCAGAAATC-3′ Reverse: 5′-GAGAAATCGATGACAGCGCC-3′

*TNF-α*	*Homo sapiens*	Forward: 5′-CTGAACTTCGGGGTGATCGG-3′ Reverse: 5′-GGCTTGTCACTCGAATTTTGAGA-3′

*iNOS*	*Homo sapiens*	Forward: 5′-TTCAAGACCAAATTCCACCAC-3′ Reverse: 5′-ATTCTGCTGCTTGCTGAGGT-3′

*IL-6*	*Homo sapiens*	Forward: 5′-ACTCACCTCTTCAGAACGAATTG-3′ Reverse: 5′-CCATCTTTGGAAGGTTCAGGTTG-3′

*CD86*	*Homo sapiens*	Forward: 5′- ATGGACCCCAGATGCACCA-3′ Reverse: 5′- CTGTGCCCAAATAGTGCTCG-3′

*ARG1*	*Homo sapiens*	Forward: 5′-GCCCTTTGCTGACATCCTA-3′ Reverse: 5′-CACCAGGCTGATTCTTCCGT-3′

*GAPDH*	*Homo sapiens*	Forward: 5′-TCAGCAATGCCTCCTGCAC-3′ Reverse: 5′-TCTGGGTGGCAGTGATGGC-3′

## Data Availability

The data that support the findings of this study are available from the corresponding author upon reasonable request.
